# Supplementary material for: Inositol pyrophosphates promote the interaction of SPX domains with the coiled-coil motif of PHR transcription factors to regulate plant phosphate homeostasis
Source: Nat Commun. 2021 Jan 15;12:384. doi: 10.1038/s41467-020-20681-4 (PMC7810988; doi:10.1038/s41467-020-20681-4)
Supplement: Supplementary file 1 — Supplementary Information [file 41467_2020_20681_MOESM1_ESM.pdf]

Supplementary Information for "Inositol pyrophosphates promote the interaction of SPX domains with the coiled-coil motif of PHR transcription factors to regulate plant phosphate homeostasis."

Martina K. Ried[1,*,%], Rebekka Wild[1,*,#], Jinsheng Zhu[1], Joka Pipercevic[2], Kristina Sturm[1], Larissa Broger[1], Robert K. Harmel[3], Luciano A. Abriata[4], Ludwig A. Hothorn[5,&], Dorothea Fiedler[3], Sebastian Hiller[2], Michael Hothorn[1,§]

ORCID IDs: MKR: 0000-0003-1582-5016, RW: 0000-0003-2025-7228. JZ: 0000-0002-8131-1876, JP: 0000-0002-3316-0268, KS: 0000-0003-1653-5168, LB: 0000-0002-1303-9778, RKH: 0000-0003-4413-7730, LAA: 0000-0003-3087-8677, LAH: 0000-0002-5162-1486, DF: 0000-0002-0798-946X, SH: 0000-0002-6709-4684, MH 0000-0002-3597-5698

[1]Structural Plant Biology Laboratory, Department of Botany and Plant Biology, University of Geneva, 1211 Geneva, Switzerland.

[2]Biozentrum Basel, 4056 Basel, Switzerland

[3]Leibniz-Forschungsinstitut für Molekulare Pharmakologie, 13125 Berlin, Germany & Department of Chemistry, Humboldt-Universität zu Berlin, 12489 Berlin, Germany.

[4]Protein production and structure Core Facility, EPFL, 1015 Lausanne, Switzerland

[5]Institute of Biostatistics, Leibniz University, 30419 Hannover, Germany.

[*]contributed equally

[%]Present address: Leibniz Institute of Plant Biochemistry, 06120 Halle, Germany.

[#]Present address: Institut de Biologie Structurale (IBS), 38044 Grenoble, France.

[&]retired

[§]To whom correspondence should be addressed. Email: michael.hothorn@unige.ch

Supplementary Table 1 – Stable transgenic *A. thaliana* lines.

Promoter	N-terminal tag	Gene	Genetic background	Selection
pPHR1	FLAG	*PHR1*	*phr1-3*	*p35S:mCherry*
pPHR1	FLAG	$PHR1^{K325A}$	*phr1-3*	*p35S:mCherry*
pPHR1	FLAG	$PHR1^{H328A}$	*phr1-3*	*p35S:mCherry*
pPHR1	FLAG	$PHR1^{R335A}$	*phr1-3*	*p35S:mCherry*
pPHR1	FLAG	$PHR1^{K325A,\ R335A}$	*phr1-3*	*p35S:mCherry*
pPHR1	FLAG	$PHR1^{K325A\ H328A\ R335A}$	*phr1-3*	*p35S:mCherry*
pPHR1	eGFP	*PHR1*	*phr1 phl1*	hygromycin
pPHR1	eGFP	$PHR1^{K325A\ H328A\ R335A}$	*phr1 phl1*	hygromycin

Supplementary Table 2 – Yeast strains.

Plasmids for yeast transformation have been generated via Gibson cloning[67]. Mutations targeting AtPHR1 K325, H328 and R335 were introduced by site-directed mutagenesis PCR[68].

**Strain**	**Plasmids**	**Genotype**
TATA	untransformed	gal4::loxP-kanMX-loxP/Gal4D, ade2 trp1-901/ade2-101::loxP-kanMX-loxP, leu2-3,112/ leu2-3,-112, his3D200/ his3D200, LYS2/lys2::(lexAop)4-HIS3, ura3-52::URA3(lexAop)8-lacZ/ ura3-52 URA3::UASGAL1-LacZ
TATA	$pB29{:}AtSPX1^{1\text{-}252}$<br>$pP6{:}AtPHR1^{1\text{-}409}$	gal4::loxP-kanMX-loxP/Gal4D, ade2 trp1-901/ade2-101::loxP-kanMX-loxP, leu2-3,112/ leu2-3,-112, his3D200/ his3D200, LYS2/lys2::(lexAop)4-HIS3, ura3-52::URA3(lexAop)8-lacZ/ ura3-52 URA3::UASGAL1-LacZ, pB29-pADH1-AtSPX1-LexA-tADH1-TRP1-TetR, pP6-pADH1-Gal4_AD-AtPHR_1-1227-tADH1-LEU2-ampR
TATA	$pB29{:}AtSPX1^{1\text{-}252}$<br>$pP6{:}AtPHR1^{226\text{-}360}$	gal4::loxP-kanMX-loxP/Gal4D, ade2 trp1-901/ade2-101::loxP-kanMX-loxP, leu2-3,112/ leu2-3,-112, his3D200/ his3D200, LYS2/lys2::(lexAop)4-HIS3, ura3-52::URA3(lexAop)8-lacZ/ ura3-52 URA3::UASGAL1-LacZ, pB29-pADH1-AtSPX1-LexA-tADH1-TRP1-TetR, pP6-pADH1-Gal4_AD-AtPHR_678-1080-tADH1-LEU2-ampR
TATA	$pB29{:}AtSPX1^{1\text{-}252}$<br>$pP6{:}AtPHR1^{280\text{-}360}$	gal4::loxP-kanMX-loxP/Gal4D, ade2 trp1-901/ade2-101::loxP-kanMX-loxP, leu2-3,112/ leu2-3,-112, his3D200/ his3D200, LYS2/lys2::(lexAop)4-HIS3, ura3-52::URA3(lexAop)8-lacZ/ ura3-52 URA3::UASGAL1-LacZ, pB29-pADH1-AtSPX1-LexA-tADH1-TRP1-TetR, pP6-pADH1-Gal4_AD-PHR_840-1080-tADH1-LEU2-ampR
TATA	$pB29{:}AtSPX1^{1\text{-}252}$<br>$pP6{:}AtPHR1^{300\text{-}360}$	gal4::loxP-kanMX-loxP/Gal4D, ade2 trp1-901/ade2-101::loxP-kanMX-loxP, leu2-3,112/ leu2-3,-112, his3D200/ his3D200, LYS2/lys2::(lexAop)4-HIS3, ura3-52::URA3(lexAop)8-lacZ/ ura3-52 URA3::UASGAL1-LacZ, pB29-pADH1-AtSPX1-LexA-tADH1-TRP1-TetR, pP6-pADH1-Gal4_AD-PHR_900-1080-tADH1-LEU2-ampR
TATA	$pB29{:}AtSPX1^{1\text{-}252}$<br>$pP6{:}AtPHR1^{280\text{-}353}$	gal4::loxP-kanMX-loxP/Gal4D, ade2 trp1-901/ade2-101::loxP-kanMX-loxP, leu2-3,112/ leu2-3,-112, his3D200/ his3D200, LYS2/lys2::(lexAop)4-HIS3, ura3-52::URA3(lexAop)8-lacZ/ ura3-52 URA3::UASGAL1-LacZ, pB29-pADH1-AtSPX1-LexA-tADH1-TRP1-TetR, pP6-pADH1-Gal4_AD-PHR_840-1059-tADH1-LEU2-ampR
TATA	$pB29{:}AtSPX1^{1\text{-}252}$<br>$pP6{:}AtPHR1^{280\text{-}342}$	gal4::loxP-kanMX-loxP/Gal4D, ade2 trp1-901/ade2-101::loxP-kanMX-loxP, leu2-3,112/ leu2-3,-112, his3D200/ his3D200,

		LYS2/lys2::(lexAop)4-HIS3, ura3-52::URA3(lexAop)8-lacZ/ ura3-52 URA3::UASGAL1-LacZ, pB29-pADH1-AtSPX1-LexA-tADH1-TRP1-TetR, pP6-pADH1-Gal4_AD-PHR_840-1026-tADH1-LEU2-ampR
TATA	*pB29:AtSPX1$^{1-252}$*<br>*pP6:AtPHR1$^{1-225}$*	gal4::loxP-kanMX-loxP/Gal4D, ade2 trp1-901/ade2-101::loxP-kanMX-loxP, leu2-3,112/ leu2-3,-112, his3D200/ his3D200, LYS2/lys2::(lexAop)4-HIS3, ura3-52::URA3(lexAop)8-lacZ/ ura3-52 URA3::UASGAL1-LacZ, pB29-pADH1-AtSPX1-LexA-tADH1-TRP1-TetR, pP6-pADH1-Gal4_AD-PHR_1-675-tADH1-LEU2-ampR
TATA	*pB29:AtSPX1$^{1-252}$*<br>*pP6:AtPHR1$^{360-409}$*	gal4::loxP-kanMX-loxP/Gal4D, ade2 trp1-901/ade2-101::loxP-kanMX-loxP, leu2-3,112/ leu2-3,-112, his3D200/ his3D200, LYS2/lys2::(lexAop)4-HIS3, ura3-52::URA3(lexAop)8-lacZ/ ura3-52 URA3::UASGAL1-LacZ, pB29-pADH1-AtSPX1-LexA-tADH1-TRP1-TetR, pP6-pADH1-Gal4_AD-PHR_1080-1227-tADH1-LEU2-ampR
TATA	*pB29 AtSPX2$^{1-287}$*<br>*pP6:AtPHR1$^{226-360}$*	gal4::loxP-kanMX-loxP/Gal4D, ade2 trp1-901/ade2-101::loxP-kanMX-loxP, leu2-3,112/ leu2-3,-112, his3D200/ his3D200, LYS2/lys2::(lexAop)4-HIS3, ura3-52::URA3(lexAop)8-lacZ/ ura3-52 URA3::UASGAL1-LacZ, pB29-pADH1-AtSPX2_1-861-LexA-tADH1-TRP1-TetR, pP6-pADH1-Gal4_AD-AtPHR_678-1080-tADH1-LEU2-ampR
TATA	*pB29:AtSPX3$^{1-245}$*<br>*pP6:AtPHR1$^{226-360}$*	gal4::loxP-kanMX-loxP/Gal4D, ade2 trp1-901/ade2-101::loxP-kanMX-loxP, leu2-3,112/ leu2-3,-112, his3D200/ his3D200, LYS2/lys2::(lexAop)4-HIS3, ura3-52::URA3(lexAop)8-lacZ/ ura3-52 URA3::UASGAL1-LacZ, pB29-pADH1-AtSPX3_1-735-LexA-tADH1-TRP1-TetR, pP6-pADH1-Gal4_AD-AtPHR_678-1080-tADH1-LEU2-ampR
TATA	*pB29:AtSPX4$^{1-318}$*<br>*pP6:AtPHR1$^{226-360}$*	gal4::loxP-kanMX-loxP/Gal4D, ade2 trp1-901/ade2-101::loxP-kanMX-loxP, leu2-3,112/ leu2-3,-112, his3D200/ his3D200, LYS2/lys2::(lexAop)4-HIS3, ura3-52::URA3(lexAop)8-lacZ/ ura3-52 URA3::UASGAL1-LacZ, pB29-pADH1-AtSPX4_1-954-LexA-tADH1-TRP1-TetR, pP6-pADH1-Gal4_AD-AtPHR_678-1080-tADH1-LEU2-ampR
TATA	*pB29:AtSPX1$^{1-252\ PBC}$*<br>*pP6:AtPHR1$^{226-360}$*	gal4::loxP-kanMX-loxP/Gal4D, ade2 trp1-901/ade2-101::loxP-kanMX-loxP, leu2-3,112/ leu2-3,-112, his3D200/ his3D200, LYS2/lys2::(lexAop)4-HIS3, ura3-52::URA3(lexAop)8-lacZ/ ura3-52 URA3::UASGAL1-LacZ, pB29-pADH1-AtSPX1_Y25F_K29A_K140A-LexA-tADH1-TRP1-TetR, pP6-pADH1-Gal4_AD-PHR_678-1080-tADH1-LEU2-ampR
TATA	*pB29:AtSPX1$^{1-252\ KSC}$*<br>*pP6:AtPHR1$^{226-360}$*	gal4::loxP-kanMX-loxP/Gal4D, ade2 trp1-901/ade2-101::loxP-kanMX-loxP, leu2-3,112/ leu2-3,-112, his3D200/ his3D200, LYS2/lys2::(lexAop)4-HIS3, ura3-52::URA3(lexAop)8-lacZ/ ura3-52 URA3::UASGAL1-LacZ, pB29-pADH1-AtSPX1_K136A_K139A_K143A-LexA-tADH1-TRP1-TetR, pP6-pADH1-Gal4_AD-PHR_678-1080-tADH1-LEU2-ampR
TATA	*pB29:AtSPX1$^{1-252\ K81A}$*<br>*pP6:AtPHR1$^{226-360}$*	gal4::loxP-kanMX-loxP/Gal4D, ade2 trp1-901/ade2-101::loxP-kanMX-loxP, leu2-3,112/ leu2-3,-112, his3D200/ his3D200, LYS2/lys2::(lexAop)4-HIS3, ura3-52::URA3(lexAop)8-lacZ/ ura3-52 URA3::UASGAL1-LacZ, pB29-pADH1-AtSPX1_K81A-LexA-tADH1-TRP1-TetR, pP6-pADH1-Gal4_AD-PHR_678-1080-tADH1-LEU2-ampR
TATA	*pB29:AtSPX1$^{1-252}$*<br>*pP6:AtPHR1$^{226-360\ K308A}$*	gal4::loxP-kanMX-loxP/Gal4D, ade2 trp1-901/ade2-101::loxP-kanMX-loxP, leu2-3,112/ leu2-3,-112, his3D200/ his3D200, LYS2/lys2::(lexAop)4-HIS3, ura3-52::URA3(lexAop)8-lacZ/ ura3-52 URA3::UASGAL1-LacZ, pB29-pADH1-AtSPX1-LexA-tADH1-TRP1-TetR, pP6-pADH1-Gal4_AD-PHR_678-1080_K308A-tADH1-LEU2-ampR
TATA	*pB29:AtSPX1$^{1-252}$*<br>*pP6:AtPHR1$^{226-360\ R318A}$*	gal4::loxP-kanMX-loxP/Gal4D, ade2 trp1-901/ade2-101::loxP-kanMX-loxP, leu2-3,112/ leu2-3,-112, his3D200/ his3D200, LYS2/lys2::(lexAop)4-HIS3, ura3-52::URA3(lexAop)8-lacZ/ ura3-52 URA3::UASGAL1-LacZ, pB29-pADH1-AtSPX1-LexA-tADH1-TRP1-TetR, pP6-pADH1-Gal4_AD-PHR_678-1080_R318A-tADH1-

		LEU2-ampR
TATA	*pB29:AtSPX1*$^{1-252}$<br>*pP6:AtPHR1*$^{226-360\ K325A}$	gal4::loxP-kanMX-loxP/Gal4D, ade2 trp1-901/ade2-101::loxP-kanMX-loxP, leu2-3,112/ leu2-3,-112, his3D200/ his3D200, LYS2/lys2::(lexAop)4-HIS3, ura3-52::URA3(lexAop)8-lacZ/ ura3-52 URA3::UASGAL1-LacZ, pB29-pADH1-AtSPX1-LexA-tADH1-TRP1-TetR, pP6-pADH1-Gal4_AD-PHR_678-1080_K325A-tADH1-LEU2-ampR
TATA	*pB29:AtSPX1*$^{1-252}$<br>*pP6:AtPHR1*$^{226-360\ H328A}$	gal4::loxP-kanMX-loxP/Gal4D, ade2 trp1-901/ade2-101::loxP-kanMX-loxP, leu2-3,112/ leu2-3,-112, his3D200/ his3D200, LYS2/lys2::(lexAop)4-HIS3, ura3-52::URA3(lexAop)8-lacZ/ ura3-52 URA3::UASGAL1-LacZ, pB29-pADH1-AtSPX1-LexA-tADH1-TRP1-TetR, pP6-pADH1-Gal4_AD-PHR_678-1080_H328A-tADH1-LEU2-ampR
TATA	*pB29:AtSPX1*$^{1-252}$<br>*pP6:AtPHR1*$^{226-360\ R335A}$	gal4::loxP-kanMX-loxP/Gal4D, ade2 trp1-901/ade2-101::loxP-kanMX-loxP, leu2-3,112/ leu2-3,-112, his3D200/ his3D200, LYS2/lys2::(lexAop)4-HIS3, ura3-52::URA3(lexAop)8-lacZ/ ura3-52 URA3::UASGAL1-LacZ, pB29-pADH1-AtSPX1-LexA-tADH1-TRP1-TetR, pP6-pADH1-Gal4_AD-PHR_678-1080_R335A-tADH1-LEU2-ampR
TATA	*pB29:AtSPX1*$^{1-252}$<br>*pP6:AtPHR1*$^{226-360\ K325A\ R335A}$	gal4::loxP-kanMX-loxP/Gal4D, ade2 trp1-901/ade2-101::loxP-kanMX-loxP, leu2-3,112/ leu2-3,-112, his3D200/ his3D200, LYS2/lys2::(lexAop)4-HIS3, ura3-52::URA3(lexAop)8-lacZ/ ura3-52 URA3::UASGAL1-LacZ, pB29-pADH1-AtSPX1-LexA-tADH1-TRP1-TetR, pP6-pADH1-Gal4_AD-PHR_678-1080_ K325AR335A -tADH1-LEU2-ampR
TATA	*pB29:AtSPX1*$^{1-252}$<br>*pP6:AtPHR1*$^{226-360\ K325A\ H328A\ R335A}$	gal4::loxP-kanMX-loxP/Gal4D, ade2 trp1-901/ade2-101::loxP-kanMX-loxP, leu2-3,112/ leu2-3,-112, his3D200/ his3D200, LYS2/lys2::(lexAop)4-HIS3, ura3-52::URA3(lexAop)8-lacZ/ ura3-52 URA3::UASGAL1-LacZ, pB29-pADH1-AtSPX1-LexA-tADH1-TRP1-TetR, pP6-pADH1-Gal4_AD-PHR_678-1080_K325AH328AR335A-tADH1-LEU2-ampR
TATA	*pB29:AtSPX1*$^{1-252}$<br>*pP6:AtPHR1*$^{226-360\ R340A}$	gal4::loxP-kanMX-loxP/Gal4D, ade2 trp1-901/ade2-101::loxP-kanMX-loxP, leu2-3,112/ leu2-3,-112, his3D200/ his3D200, LYS2/lys2::(lexAop)4-HIS3, ura3-52::URA3(lexAop)8-lacZ/ ura3-52 URA3::UASGAL1-LacZ, pB29-pADH1-AtSPX1-LexA-tADH1-TRP1-TetR, pP6-pADH1-Gal4_AD-PHR_678-1080_R340A-tADH1-LEU2-ampR
L40	untransformed	MATa ade2 trp1-901 leu2-3,112 lys2-801am his3D200 lys2::(lexAop)4-HIS3, ura3-52::URA3 (lexAop)8-lacZa
L40<br>ΔVIP1	untransformed	MATa ade2 trp1-901 leu2-3,112 lys2-801am his3D200 lys2::(lexAop)4-HIS3 ura3-52::URA3 (lexAop)8-lacZa vip1::natNT2
L40<br>ΔKCS1	untransformed	MATa ade2 trp1-901 leu2-3,112 lys2-801am his3D200 lys2::(lexAop)4-HIS3, ura3-52::URA3 (lexAop)8-lacZa kcs1::natNT2
L40	*pB29:AtSPX1*$^{1-252}$<br>*pP6:AtPHR1*$^{226-360}$	MATa ade2 trp1-901 leu2-3,112 lys2-801am his3D200 lys2::(lexAop)4-HIS3, ura3-52::URA3 (lexAop)8-lacZa pB29-pADH1-AtSPX1-LexA-tADH1-TRP1-TetR, pP6-pADH1-Gal4_AD-AtPHR_678-1080-tADH1-LEU2-ampR
L40<br>ΔVIP1	*pB29:AtSPX1*$^{1-252}$<br>*pP6:AtPHR1*$^{226-360}$	MATa ade2 trp1-901 leu2-3,112 lys2-801am his3D200 lys2::(lexAop)4-HIS3, ura3-52::URA3 (lexAop)8-lacZa vip1::natNT2 pP6-pADH1-Gal4_ADAtPHR_678-1080-tADH1-LEU2-ampR pB29-pADH1-AtSPX1-LexA-tADH1-<br>TRP1-TetR
L40<br>ΔKCS1	*pB29:AtSPX1*$^{1-252}$<br>*pP6:AtPHR1*$^{226-360}$	MATa ade2 trp1-901 leu2-3,112 lys2-801am his3D200 lys2::(lexAop)4-HIS3, ura3-52::URA3 (lexAop)8-lacZa kcs1::natNT2 pP6-pADH1-Gal4_ADAtPHR_678-1080-tADH1-LEU2-ampR pB29-pADH1-AtSPX1-LexA-tADH1-<br>TRP1-TetR
L40	*pB29:AtBRI1*$^{828-1196}$<br>*pP6:AtBKI1*$^{1-337}$	MATa ade2 trp1-901 leu2-3,112 lys2-801am his3D200 lys2::(lexAop)4-HIS3, ura3-52::URA3 (lexAop)8-lacZa pP6-pADH1-Gal4_AD_pP6_AtBKI1_1-1011-tADH1-LEU2-ampR pB29-pADH1-

		AtBRI1_2484-3588-LexA-tADH1-TRP1-TetR
L40 ΔVIP1	*pB29:AtBRI1*$^{828-1196}$ *pP6:AtBKI1*$^{1-337}$	MATa ade2 trp1-901 leu2-3,112 lys2-801am his3D200 lys2::(lexAop)4-HIS3, ura3-52::URA3 (lexAop)8-lacZa vip1::natNT2 pP6-pADH1-Gal4_AD_pP6_AtBKI1_1-1011-tADH1-LEU2-ampR pB29-pADH1-AtBRI1_2484-3588-LexA-tADH1-TRP1-TetR
L40 ΔKCS1	*pB29:AtBRI1*$^{828-1196}$ *pP6:AtBKI1*$^{1-337}$	MATa ade2 trp1-901 leu2-3,112 lys2-801am his3D200 lys2::(lexAop)4-HIS3 ura3-52::URA3 (lexAop)8-lacZa kcs1::natNT2 pP6-pADH1-Gal4_AD_pP6_AtBKI1_1-1011-tADH1-LEU2-ampR pB29-pADH1-AtBRI1_2484-3588-LexA-tADH1-TRP1-TetR

Supplementary Table 3 – Constructs and primers.

a, Golden Gate Level 0 constructs and primers. The *PHR1* promoter and gene were amplified from *A. thaliana* gDNA, and *SPX1* was amplified from *A. thaliana* cDNA. Level 0 constructs were generated via StuI or SmaI cut-ligation into pUC Amp[69].

Name	Primer	Sequences
pPHR1.1	OutFwdProm<br>InRevProm	ACGAAGACGTTACGGGTCTCTGCGGATTTTGTAAAACTATGAATC<br>AGGAAGACGGAAAACGAATCGAATCGGAGAAAATG
pPHR1.2	InFwdProm<br>OutRevProm	ATGAAGACCGTTTTCCTTGGTCCTGGATTGC<br>ATGAAGACACCAGAGGTCTCTCAGATGTTGTCCTGCAAGAGAGAATC
PHR1.1	OutFwdGene<br>InRevGene1	AAGAAGACTCTACGGGTCTCCCACCATGGAGGCTCGTCCAGTTCATAGATCA GGTTCGAGGGACC<br>GCGAAGACTTTTGGTCCTAAAAAAGTGTGTCCAG
PHR1.2	InFwdGene1<br>InRevGene2	TTGAAGACGACCAAAAGACACTGCAAATTCCGCAACC<br>TAGAAGACGAAGCCAATTATATGCATTAGCAGG
PHR1.3	InFwdGene2<br>InRevGene3	GCGAAGACTTGGCTTCTAATATTAGATTGTG<br>TTGAAGACTCCTCTTGTTCAGATTTGGCTGCGGA
PHR1.4	InFwdGene3<br>OutRevGene	AAGAAGACCAAGAGGACAAGAAAACTGCTGATTCG<br>ATGAAGACAACAGAGGTCTCTCCTTATTATCGATTTTGGGACGC
PHR1^{K325A} a<br>*PHR1$^{K325A\ R335A}$ a*<br>*PHR1$^{K325A\ H328A\ R335A}$ a*	InFwdGene2<br>InRevKA	GCGAAGACTTGGCTTCTAATATTAGATTGTG<br>CAGAAGACTGCGCCTGTACTTCCATCTGAAGTCG
PHR1^{K325A} b	InFwdKA<br>InRevGene3	GTGAAGACACGGCGCAACTCCATGAGCAGCTCGAG<br>TTGAAGACTCCTCTTGTTCAGATTTGGCTGCGGA
PHR1^{H328A} a	InFwdGene2<br>InRevHA	GCGAAGACTTGGCTTCTAATATTAGATTGTG<br>CGGAAGACTCAGCGAGTTGCTTCTGTACTTCC
PHR1^{H328A} b	InFwdHA<br>InRevGene3	AGGAAGACCTCGCTGAGCAGCTCGAGGTATGTTC<br>TTGAAGACTCCTCTTGTTCAGATTTGGCTGCGGA
PHR1^{R335A} a	InFwdGene2<br>InRevRA	GCGAAGACTTGGCTTCTAATATTAGATTGTG<br>GTGAAGACTTTGCTTGAATCTGCAGGCAAAGG
PHR1^{R335A} b<br>*PHR1$^{K325A\ R335A}$ c*<br>*PHR1$^{K325A\ H328A\ R335A}$ c*	InFwdRA<br>InRevGene3	TAGAAGACCAAGCAAACCTGCAACTCCGAATAGAAG<br>TTGAAGACTCCTCTTGTTCAGATTTGGCTGCGGA
PHR1$^{K325A\ R335A}$ b	InFwdKA<br>InRevRA	GTGAAGACACGGCGCAACTCCATGAGCAGCTCGAG<br>GTGAAGACTTTGCTTGAATCTGCAGGCAAAGG
PHR1$^{K325A\ H328A\ R335A}$ b	InFwdKAHA<br>InRevRA	GTGAAGACACGGCGCAACTCGCTGAGCAGCTCGAG<br>GTGAAGACTTTGCTTGAATCTGCAGGCAAAGG
SPX1.1	OutFwdGene<br>InRevGene1	AAGAAGACCATACGGGTCTCGCACCATGAAGTTTGGTAAGAGTC<br>TCGAAGACGTCCTCTAACAATTGGATGAAATTG
SPX1.2	InFwdGene1<br>InRevGene2	CCGAAGACTAGAGGACGAGTTGGAGAAATTCAAC<br>TCGAAGACTGGAGACTCTCCATGAACTTATGC
SPX1.3	InFwdGene2<br>OutRevGene	TCGAAGACAGTCTCCATATGAAGAGCACAATCGC<br>AGGAAGACTGCAGAGGTCTCACCTTTTTGGCTTCTTGCTCCAAC

b, Level I, II & III constructs. Level 1 and level 3 constructs were generated via BpiI cut-ligation, and level 2 constructs via BsaI cut-ligation[69].

Name	Assembly	Purpose
LI BpiI pPHR1	pPHR1.1 + pPHR1.2	Cloning

LI BpiI PHR1	PHR1.1 + PHR1.2 + PHR1.3 + PHR1.4	Cloning
LI BpiI PHR1^{K}	PHR1.1 + PHR1.2 + PHR1^{K325A} a + PHR1^{K325A} b + PHR1.4	Cloning
LI BpiI PHR1^{H}	PHR1.1 + PHR1.2 + PHR1^{H328A} a + PHR1^{H328A} b + PHR1.4	Cloning
LI BpiI PHR1^{R}	PHR1.1 + PHR1.2 + PHR1^{R335A} a + PHR1^{R335A} b + PHR1.4	Cloning
LI BpiI PHR1^{KR}	PHR1.1 + PHR1.2 + PHR1$^{K325A\ R335A}$ a + PHR1$^{K325A\ R335A}$ b + PHR1$^{K325A\ R335A}$ c + PHR1.4	Cloning
LI BpiI PHR1$^{KHR/A}$	PHR1.1 + PHR1.2 + PHR1$^{K325A\ H328A\ R335A}$ a + PHR1$^{K325A\ H328A\ R335A}$ b + PHR1$^{K325A\ H328A\ R335A}$ c + PHR1.4	Cloning
LI SPX1	SPX1.1 + SPX1.2 + SPX1.3	Cloning
LII R5-6 pPHR1:FLAG-PHR1	LI A-B pPHR1 + LI FLAG B-C + LI C-D PHR1 + LI dy D-E + LI E-F nos-T + LI dy F-G	Cloning
LII R5-6 pPHR1:FLAG-PHR1^{K}	LI A-B pPHR1 + LI FLAG B-C + LI C-D PHR1^{K} + LI dy D-E + LI E-F nos-T + LI dy F-G	Cloning
LII R5-6 pPHR1:FLAG-PHR1^{H}	LI A-B pPHR1 + LI FLAG B-C + LI C-D PHR1^{H} + LI dy D-E + LI E-F nos-T + LI dy F-G	Cloning
LII R5-6 pPHR1:FLAG-PHR1^{R}	LI A-B pPHR1 + LI FLAG B-C + LI C-D PHR1^{R} + LI dy D-E + LI E-F nos-T + LI dy F-G	Cloning
LII R5-6 pPHR1:FLAG-PHR1^{KR}	LI A-B pPHR1 + LI FLAG B-C + LI C-D PHR1^{KR} + LI dy D-E + LI E-F nos-T + LI dy F-G	Cloning
LII R5-6 pPHR1:FLAG-PHR1$^{KHR/A}$	LI A-B pPHR1 + LI FLAG B-C + LI C-D PHR1^{KHR} + LI dy D-E + LI E-F nos-T + LI dy F-G	Cloning
LII F1-2 p35S:mCherry	LI A-B p35S + LI dy B-C + LI C-D mCherry + LI dy D-E + LI E-F 35S-T + LI dy F-G	Cloning / *N. benthamiana* transformation
LII F1-2 p35S:mCherry-SPX1	LI A-B p35S + LI mCherry B-C + LI C-D SPX1 + LI dy D-E + LI E-F nos-T + LI dy F-G	*N. benthamiana* transformation
LII F1-2 p35S:GFP-PHR1	LI A-B p35S + LI GFP B-C + LI C-D PHR1 + LI dy D-E + LI E-F nos-T + LI dy F-G	*N. benthamiana* transformation
LII F1-2 p35S:GFP-PHR1$^{KHR/A}$	LI A-B p35S + LI GFP B-C + LI C-D PHR1^{KHR} + LI dy D-E + LI E-F nos-T + LI dy F-G	*N. benthamiana* transformation
LIIIβ fin p35S:mCherry pPHR1:FLAG-PHR1	LII p35S:mCherry 1-2 + LII ins 2-3 + LII dy 3-4 + LII ins 4-5 + LII F LII pPHR1:FLAG-PHR1 5-6	*A. thaliana* transformation
LIIIβ fin p35S:mCherry pPHR1:FLAG-PHR1^{K}	LII p35S:mCherry 1-2 + LII ins 2-3 + LII dy 3-4 + LII ins 4-5 + LII F LII pPHR1:FLAG-PHR1 $^{\mathbf{K}}$ 5-6	*A. thaliana* transformation
LIIIβ fin p35S:mCherry pPHR1:FLAG-PHR1^{H}	LII p35S:mCherry 1-2 + LII ins 2-3 + LII dy 3-4 + LII ins 4-5 + LII F LII pPHR1:FLAG-PHR1 $^{\mathbf{H}}$ 5-6	*A. thaliana* transformation
LIIIβ fin p35S:mCherry pPHR1:FLAG-PHR1^{R}	LII p35S:mCherry 1-2 + LII ins 2-3 + LII dy 3-4 + LII ins 4-5 + LII F LII pPHR1:FLAG-PHR1 $^{\mathbf{R}}$ 5-6	*A. thaliana* transformation
LIIIβ fin p35S:mCherry pPHR1:FLAG-PHR1^{KR}	LII p35S:mCherry 1-2 + LII ins 2-3 + LII dy 3-4 + LII ins 4-5 + LII F LII pPHR1:FLAG-PHR1 $^{\mathbf{KR}}$ 5-6	*A. thaliana* transformation
LIIIβ fin p35S:mCherry pPHR1:FLAG-PHR1$^{KHR/A}$	LII p35S:mCherry 1-2 + LII ins 2-3 + LII dy 3-4 + LII ins 4-5 + LII F LII pPHR1:FLAG-PHR1 $^{\mathbf{KHR}}$ 5-6	*A. thaliana* transformation

c, Plasmids for recombinant protein expression in *E. coli.*

Plasmids for recombinant protein expression in *E. coli* have been generated via Gibson cloning[67]. Mutations targeting AtPHR1 K325, H328 and R335 were introduced by site-directed mutagenesis PCR[68].

**Vector**	**Construct**	**Purpose**
pMH_HT	*AtPHR1*$^{222\text{-}358}$	OmniSEC, GCI, EMSA

pMH_HT	*AtPHR1*$^{222\text{-}358\ Olig1}$	OmniSEC, GCI, EMSA
pMH_HT	*AtPHR1*$^{222\text{-}358\ Olig2}$	OmniSEC, GCI, EMSA
pMH_HT	*AtPHR1*$^{280-360}$	OmniSEC
pMH_HT	*AtPHR1*$^{280-360\ Olig1}$	OmniSEC
pMH_HT	*AtPHR1*$^{280-360\ Olig2}$	OmniSEC
pMH_HT	*AtPHR1*$^{280\text{-}360\ K325A,\ H328A,\ R335A}$	OmniSEC
pMH_HSgb1T	*OsPHR2*$^{1-426}$	OmniSEC, GCI, ITC
pMH_HSgb1T	*OsPHR2*$^{1-426\ KHR/A}$	OmniSEC, GCI, ITC
pMH_Hssumo	*OsSPX4*$^{1\text{-}321}$	ITC
pMH_Hssumo	*AtPHR1*$^{280-360}$	Crystallisation

d, Characterisation of T-DNA mutants.

**Name**	**5'-3' Sequence**
LBb1.3	ATTTTGCCGATTTCGGAAC
LP_PHR1	GAGAGACCTCACACGCACTTC
RP_PHR1	CTTTCTGGCGAACCTGTAGTG
phl1-LP	GTGGAGACGTTTCTGCACTTC
phl1-RP	TCCCACAATCCAAATTCAGAG

e, Gene expression analysis.

**Gene identifier**	**Name**	**Sequence**
At3g18780	Actin2_F	AGTGGTCGTACAACCGGTATTGT
	Actin2_R	GATGGCATGGAGGAAGAGAGAAAC
At4g28610	PHR1_F	GTTCAGCAGCAACCTTCTCC
	PHR1_R	GCTCTTTCACTACCGCCAAG
At1g23010	LPR1_F	CCGGGCTATGTCTACCATTGTCAC
	LPR1_R	GCACCATCAAAACTTCGCAGAGATCG
At3g52820	ACP5_F	CAGTTTCTAACTAGTGGTGCTGGA
	ACP5_R	GCTTGGGATTGATGGTCACT
At3g09922	IPS1_F	TGAAGACTGCAGAAGGCTGA
	IPS1_R	CGAAGCTTGCCAAAGGATAG
At2g11810	MGD3_F	AGAGGCCGGTTTAATGGAGT
	MGD3_R	CATCAGAGGATGCACGCTAA
At1g52940	PAP5_F	TCGAACCCGAAAGGCCAAGCGGTGC
	PAP5_R	GCGCTTGGTTCCACAAACCGGCCGTA
At2g38940	PHT1;4_F	CCTCGGTCGTATTTATTACCACG
	PHT1;4_R	CCATCACAGCTTTTGGCTCATG
At5g20150	SPX1_F	CGGGTTTTGAAGGAGATCAG
	SPX1_R	GCGGCAATGAAAACACACTA

f, Primers used for cloning PHR1 into the pH7m34GW vector.

**Name**	**5'-3' Sequence**
PHR1_B2F	GGGGACAGCTTTCTTGTACAAAGTGGATGAGGCTCGTCCAGTTCATAG

PHR1_B3R	GGGGACAACTTTGTATAATAAAGTTGATCAATTATCGATTTTGGGACG
eGFP_B1F	GGGGACAAGTTTGTACAAAAAAGCAGGCTTAATGGTGAGCAAGGGCGAGGAGCTG
eGFP_B2R	GGGGACCACTTTGTACAAGAAAGCTGGGTACTTGTACAGCTCGTCCATGCC
PHR1_H328A_F	CAACTCGCTGAGCAGCTCGAAATTCAAGCAAACCTGCAACTCCG
PHR1_H328A_R	AGCTGCTCAGCGAGTTGCGCCTGTACTTCCATCTGAAGTCGTAGA
PHR1_R335A_F	ATTCAAGCAAACCTGCAACTCCGAATAGAAGAACAAGG
PHR1_R335A_R	CAGGTTTGCTTGAATTTCGAGCTGCTCATGGAG
PHR1_K325A_F	GTACAGGCGCAACTCCATGAGCAGCTCAGAAATT
PHR1_K325A_R	GAGTTGCGCCTGTACTTCCATCTGAAGTCGTAGAGC

Supplementary Tabel 4 – Modified DNA oligos.

The P1BS (GNATATNC) is shown in bold.

a, IRdye end-labelled oligos for EMSA.

Name	Sequence
AtSPX1_800 _F	5'-IRD800-CAG AGA AAA AA**G GAT ATT C**TA ATT AGA AAC CTT AA**G AAT ATT C**TT TTT AAT CCC-3'
AtSPX1_800_R	5'-IRD800-GGG ATT AAA AA**G AAT ATT C**TT AAG GTT TCT AAT TA**G AAT ATC C**TT TTT TCT CTG-3'

b, Biotinylated oligos for GCI.

Name	Sequence
AtSPX1_Biotin_F	5'-Biotin-CAG AGA AAA AA**G GAT ATT C**TA ATT AGA AAC CTT AA**G AAT ATT C**TT TTT AAT CCC-3'
AtSPX1_R	5'-GGG ATT AAA AA**G AAT ATT** CTT AAG GTT TCT AAT TA**G AAT ATC** CTT TTT TCT CTG-3'
OsIPS1_Biotin_F	5'-Biotin-TAA TGC TCG CC**G CAT ATC** CTT TGG TAG ATA-3'
OsIPS1_R	5'-TAT CTA CCA AAG **GAT ATG C**GG CGA GCA TTA-3'

Supplementary Table 5 – Crystallographic data collection and refinement statistics.

PDB-ID	**AtPHR1$^{280\text{-}360}$ form1** **6TO5**	**AtPHR1$^{280\text{-}360}$ form2** **6TO9**	**AtPHR1$^{280\text{-}360}$ form3** **6TOC**
**Data collection**			
Space group	$P\ 6_1\ 2\ 2$	$P\ 3_2\ 2\ 1$	$P\ 4_2$
Cell dimensions			
a, *b*, *c* (Å)	70.4, 70,4, 148.88	70.05, 70.05, 80.17	31.52, 31.52, 81.60
α, β, γ (°)	90, 90, 120	90, 90, 120	90, 90, 90
Resolution (Å)	47.18 – 2.38 (2.52 – 2.38)	48.37 – 2.45 (2.59 – 2.44)	31.52 – 1.85 (1.97 – 1.85)
R_{meas}#	0.189 (2.21)	0.164 (2.77)	0.073 (2.76)
CC(1/2)#	0.99 (0.69)	0.99 (0.51)	1.0 (0.40)
$I/\sigma I$#	14.85 (1.48)	15.86 (1.07)	21.41 (0.98)
Completeness (%)#	99.8 (98.9)	99.3 (96.0)	99.9 (99.3)
Redundancy#	20.8 (20.2)	19.0 (18.4)	13.5 (13.4)
Wilson B-factor#	56.7	67.8	47.1
**Refinement**			
Resolution (Å)	41.18 – 2.38	48.37 – 2.45	31.52 – 1.85
No. reflections	16,560	8,702	6,433
$R_{work}/R_{free}$$	0.22 (0.23)	0.23 (0.26)	0.21 (0.26)
No. atoms			
protein	940	952	753
solvent	18	4	27
Res. B-factors$			
protein	64.6	79.1	41.3
solvent	57.4	59.3	40.7
R.m.s deviations$			
bond lengths (Å)	0.0047	0.0049	0.012
bond angles (°)	0.89	0.98	1.61
Ramachandran plot$:			
most favored regions (%)	99.08	99.07	98.8
outliers (%)	0	0	0
MolProbity score$	1.31	1.19	1.24

*as defined in XDS[48]
+as defined phenix.refine[51] (form 1, form2) or Refmac5[52] (form 3, using twin laws h,k,l and -k, -h, -l with twin fractions of 0.5, apparent point group was P 4 2 2)
$as defined in Molprobity[53]

Supplementary References

67. Gibson, D. G. et al. Enzymatic assembly of DNA molecules up to several hundred kilobases. Nat. Methods **6**, 343–345 (2009).
68. Liu, H. & Naismith, J. H. An efficient one-step site-directed deletion, insertion, single and multiple-site plasmid mutagenesis protocol. BMC Biotechnol. **8**, 91 (2008).
69. Binder, A. et al. A modular plasmid assembly kit for multigene expression, gene silencing and silencing rescue in plants. PloS One **9**, e88218 (2014).
70. Kabsch, W. & Sander, C. Dictionary of protein secondary structure: pattern recognition of hydrogen-bonded and geometrical features. Biopolymers **22**, 2577–2637 (1983).

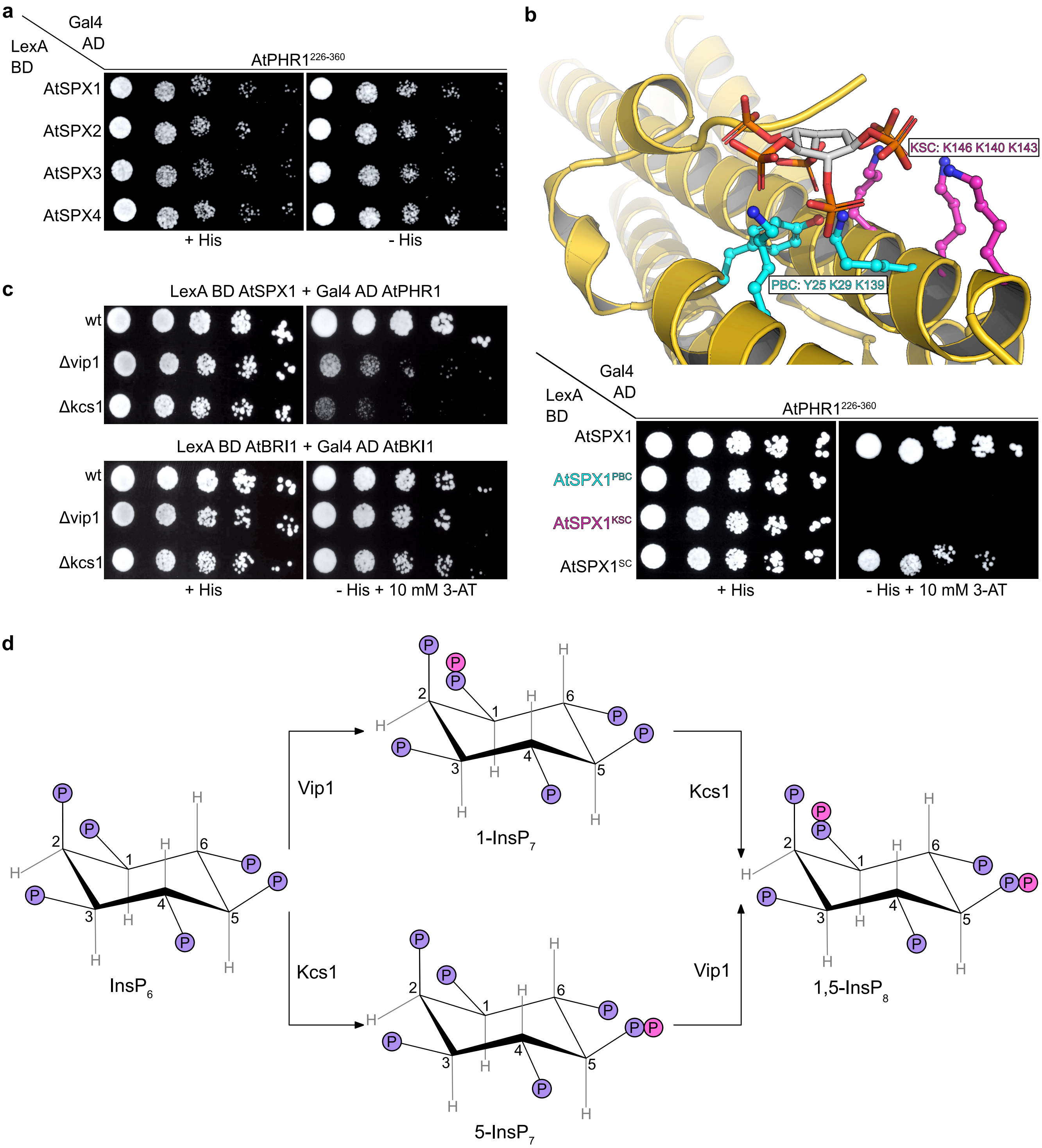

Supplementary Fig. 1 The AtPHR1 – AtSPX1 interaction in yeast is meditated by PP-InsPs.
a Yeast-two-hybrid assay. Yeast co-expressing AtPHR1$^{226\text{-}360}$ fused to the Gal4-AD (prey) and different AtSPX proteins fused to the LexA-BD (bait) were grown on selective SD medium supplemented with histidine (+ His; co-transformation control) or lacking histidine (- His; interaction assay). Shown are serial dilutions from left to right. **b** (Top panel) Homology model of an AtSPX1$^{1\text{-}182}$-$InsP_6$ complex. AtSPX1$^{1\text{-}182}$ is shown as blue ribbon diagram and side chains involved in $InsP_6$ binding are highlighted in green (PBC, phosphate binding cluster) and purple (KSC, lysine surface cluster) and depicted in bonds representation. The $InsP_6$ ligand is shown in grey (in bonds representation). (Bottom panel) Yeast co-expressing AtPHR1$^{226\text{-}360}$ fused to the Gal4-AD (prey) and different AtSPX1 versions mutated in residues involved in $InsP_6$ binding, or a structural control mutant (SC[30]) fused to the LexA-BD (bait) were grown on selective SD medium supplemented with histidine (+ His; co-transformation control) or lacking histidine and supplemented with 10 mM 3-AT(- His + 3-AT; interaction assay) to investigate the importance of the PP-InsP binding surface in AtSPX1 for the AtSPX1 – AtPHR1 interaction in yeast. **c** Yeast knock-out strains for the PP-InsP biosynthesis enzymes Vip1 or Kcs1 co-expressing either AtPHR1$^{226\text{-}360}$ fused to the Gal4-AD (prey) and AtSPX1 fused to the LexA-BD (bait) (upper panel), or AtBKI1 fused to the Gal4-AD (prey) and AtBRI1 fused to the LexA-BD (bait) (lower panel) were grown on selective SD medium supplemented with histidine (+ His; co-transformation control) or lacking histidine and supplemented with 10 mM 3-AT (- His + 3-AT; interaction assay) to investigate the importance of the availability of specific PP-InsPs for the AtSPX1-AtPHR1 interaction in yeast. **d** Schematic representation of the PP-InsP biosynthesis pathway in yeast.

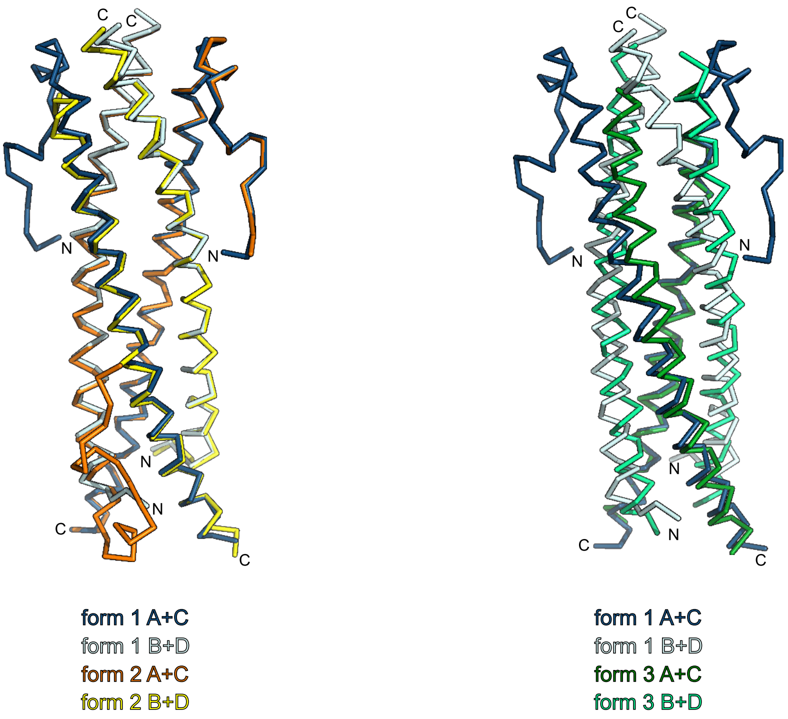

Supplementary Fig. 2 Three different AtPHR1 coiled-coil domain crystal structures all share the same tetrameric arrangement. Structural superposition (shown as C_α traces) of the four-stranded anti-parallel CC domain of AtPHR1 from crystal forms 1-3.

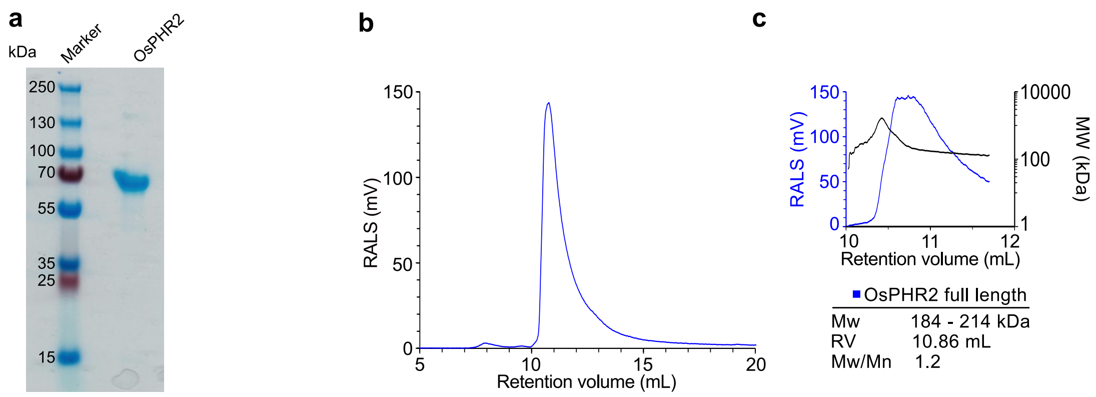

Supplementary Fig. 3 Bacterial expressed full-length OsPHR2 behaves as a tetramer in solution.

a SDS-PAGE of OsPHR2 purified from *E. coli*. **b** Analytical size exclusion chromatography traces of wild type OsPHR2. The corresponding right-angle light scattering (RALS) traces are shown in c, the molecular masses are depicted by a black line. Table summaries provide the molecular weight (Mw), retention volume (RV) and dispersity (Mw/Mn).

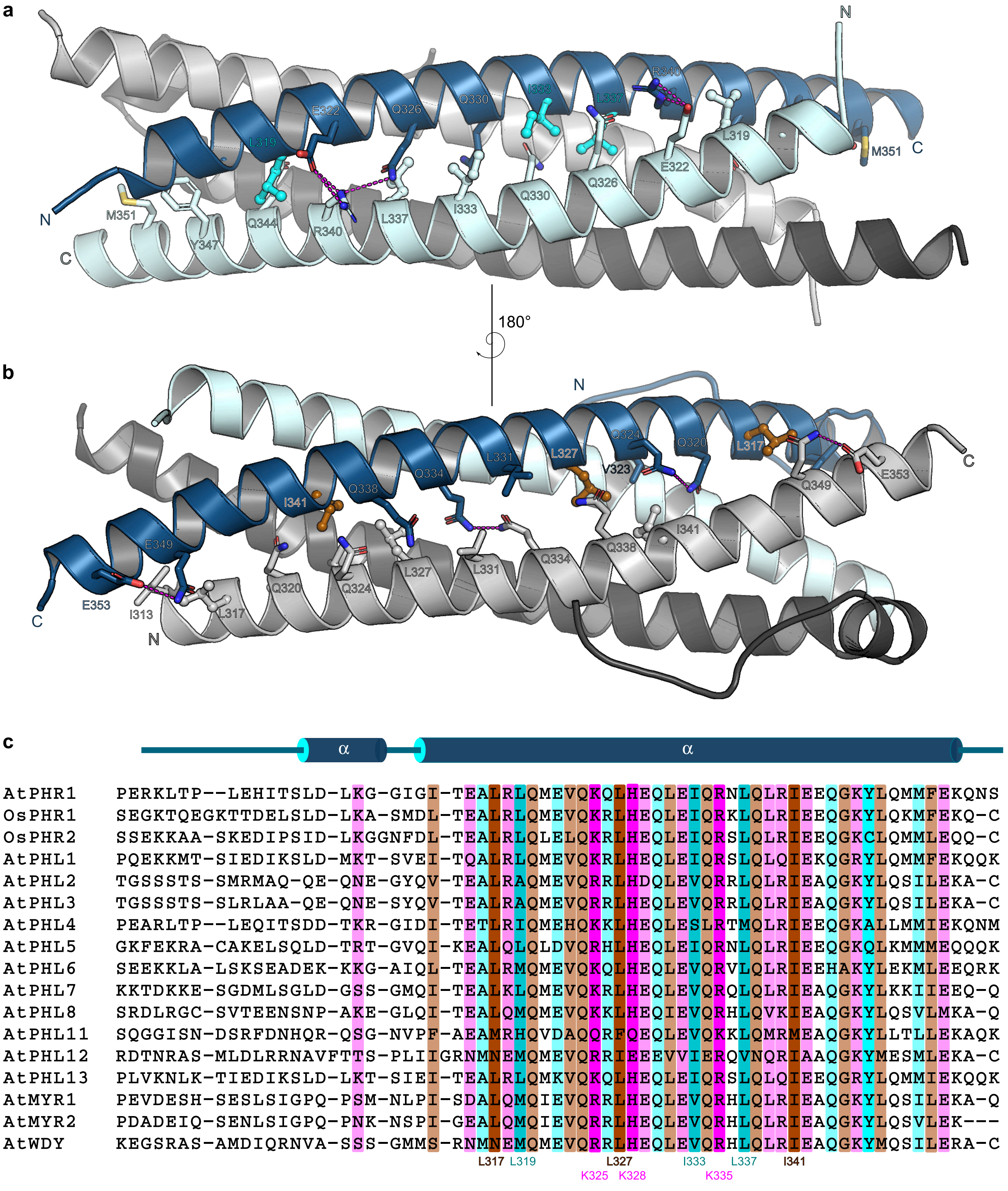

Supplementary Fig. 4 A conserved dimer- and tetramerization interface in plant MYB CC transcription factors.
a Overview of the AtPHR1 CC dimerization interface. Shown is a ribbon diagram with selected residues contributing to the dimer interface shown in bonds representation. Hydrogen bonds are indicated as dotted lines, residues mutated in the Olig 1 mutants are highlighted in cyan. **b** Overview of the tetramerization interface, with residues mutated in Olig 2 depicted in gold. **c** Structure based sequence alignment of the CC domain of plant MYB CC domain and including a secondary structure assignment calculated with the program DSSP[64]. Residues contributing to the CC dimer interface are shown in blue and cyan, to the tetramerization interface in gold and brown, respectively. The conserved basic residues on the surface of the CC domain are highlighted in magenta.

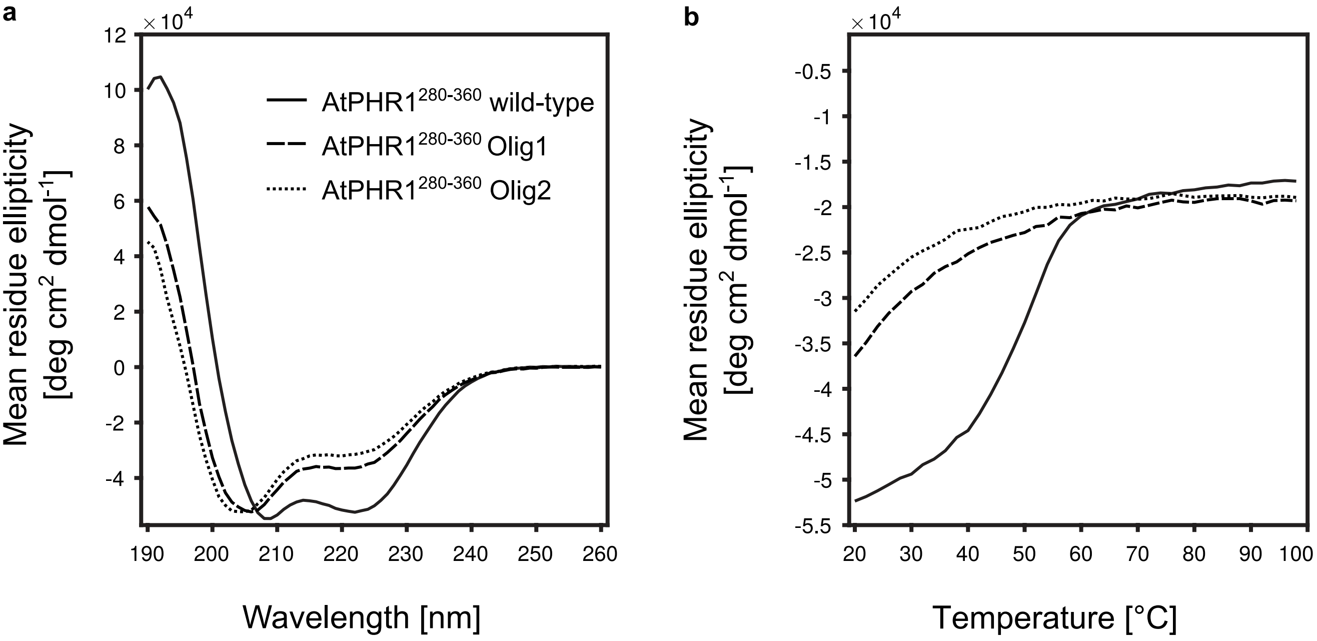

Supplementary Fig. 5 Circular Dichroism (CD) spectroscopy of wild-type and mutant AtPHR1 coiled-coil domains.
a CD spectra of AtPHR1 CC fragments from 260 nm to 190 nm, measured at 24 °C. **b** Thermal denaturation of AtPHR1 CC domain fragments. Melting curves were acquired at 222 nm with 2 °C stepwise increments in 30 intervals from 24 to 98 °C.

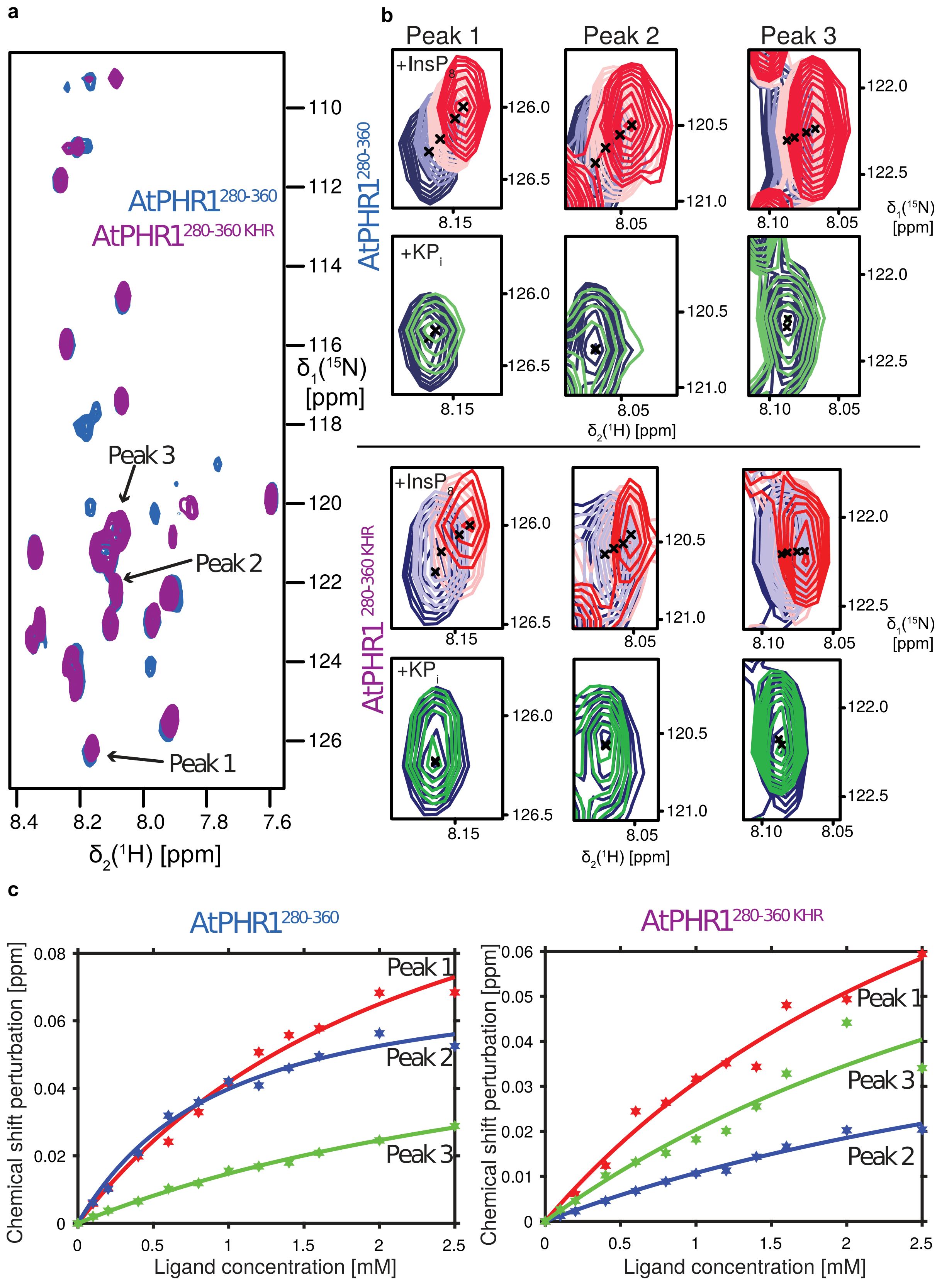

K_D (mM)	Peak 1	Peak 2	Peak 3
AtPHR1$^{280\text{-}360}$	2.2 ±1.0	0.8 ±0.3	3.8 ±1.2
AtPHR1$^{280\text{-}360\ KHR}$	2.2 ±1.2	3.1 ±0.9	3.2 ±3.4

Supplementary Fig. 6 Wild-type and mutant AtPHR1 weakly bind InsP$_8$ with a dissociation constant in the low millimolar range. **a** Comparison of 2D [^{15}N,^{1}H]-TROSY spectra of AtPHR1$^{280\text{-}360}$ (blue) and AtPHR1$^{280\text{-}360\ KHR/A}$ (purple). Black arrows indicate backbone resonance peaks 1-3 that were used to estimate an approximate dissociation constant for InsP$_8$. Both TROSY spectra were recorded with 4 scans and with 128 points in the indirect dimension. **b** Chemical shift perturbations of backbone resonance peaks 1-3 of AtPHR1$^{280\text{-}360}$ or **c** AtPHR1$^{280\text{-}360\ KHR/A}$, respectively, while titrating InsP$_8$ (top) or KP$_i$ as a negative control (bottom). The TROSY titration spectra of the protein to InsP$_8$ and KP$_i$ and InsP$_8$ were recorded with 128 points in the indirect dimension and four scans. Dark blue - 200 µM of [U-^{15}N,^{2}H]- AtPHR1$^{280\text{-}360}$ or [U-^{15}N,^{2}H]- AtPHR1$^{280\text{-}360\ KHR/A}$, light blue - 2 equivalent of InsP$_8$, purple - 6 equivalent of InsP$_8$, red - 10 equivalent InsP$_8$ and green – 80 equivalent of KP$_i$. d) Estimated dissociation constant (K_D) of AtPHR1$^{280\text{-}360}$ (right) and AtPHR1$^{280\text{-}360\ KHR/A}$ (left) by chemical shift perturbations (top) and an overview of the K_D's extracted from backbone resonance peaks 1-3 (below).

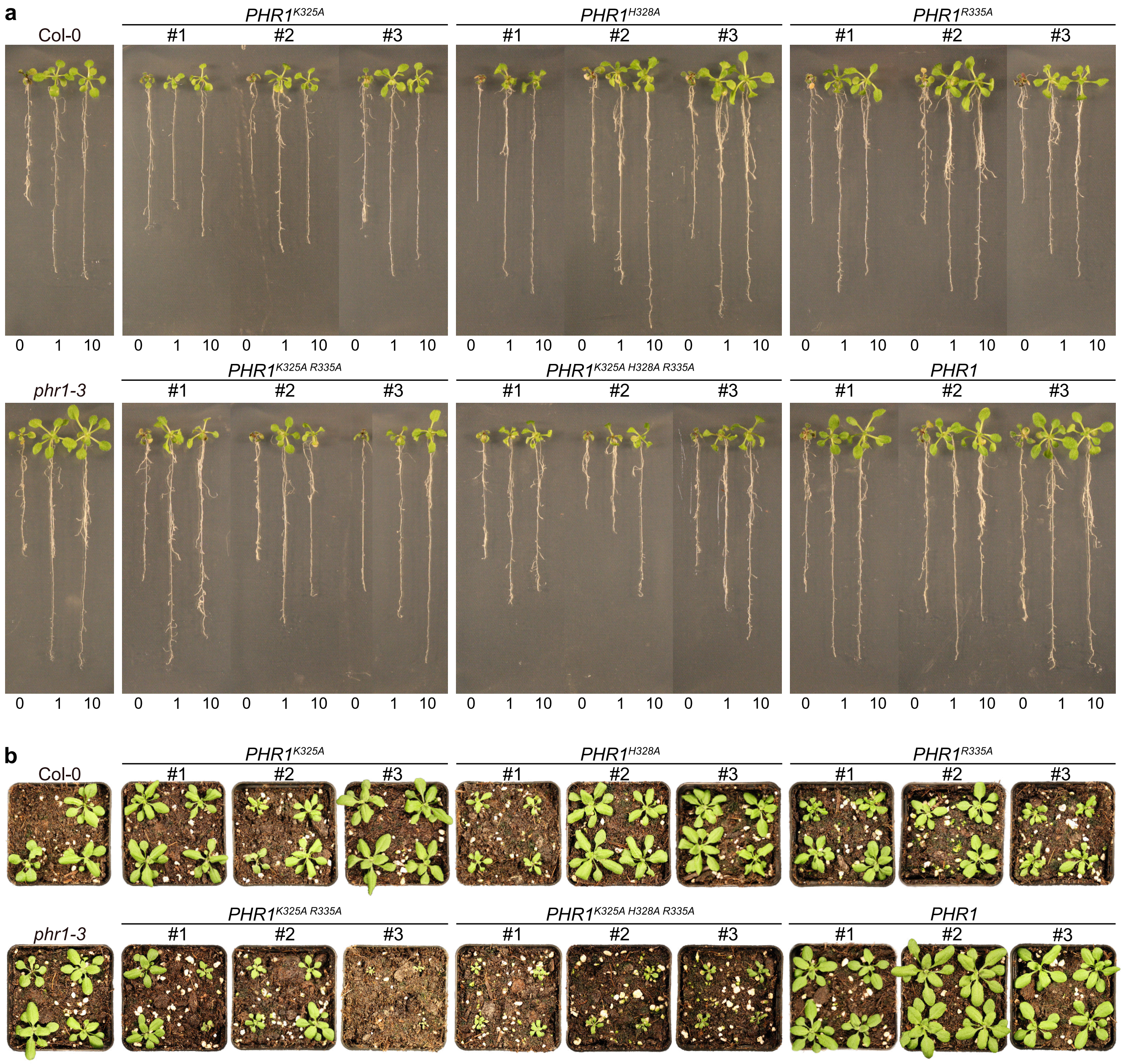

Supplementary Fig. 7 Growth phenotypes of AtPHR1 CC domain mutants that abolish interaction with AtSPX1 and impact Pi homeostasis. **a** Growth phenotype of Col-0 wild type, *phr1-3* , and seedlings of *phr1-3* complementation lines expressing FLAG-AtPHR1, FLAG-AtPHR1^{K325A}, FLAG-AtPHR1^{H328A}, FLAG-AtPHR1^{R335A}, FLAG-AtPHR1$^{K325A\ R335A}$, and FLAG-AtPHR1$^{K325A\ H328A\ R335A}$ under the control of the *AtPHR1* promoter at 14 d after germination (DAG). Seedlings were germinated and grown on vertical $^{1/2}$MS plates for 8 d, transferred to $^{1/2}$MS plates supplemented with either 0 mM, 1 mM or 10 mM Pi and grown for additional 7 d. **b** Growth phenotypes of the lines in **a**, at 21 DAG. Seedlings were germinated and grown on vertical $^{1/2}$MS plates for eight days, transferred to soil and grown for additional 14 d.

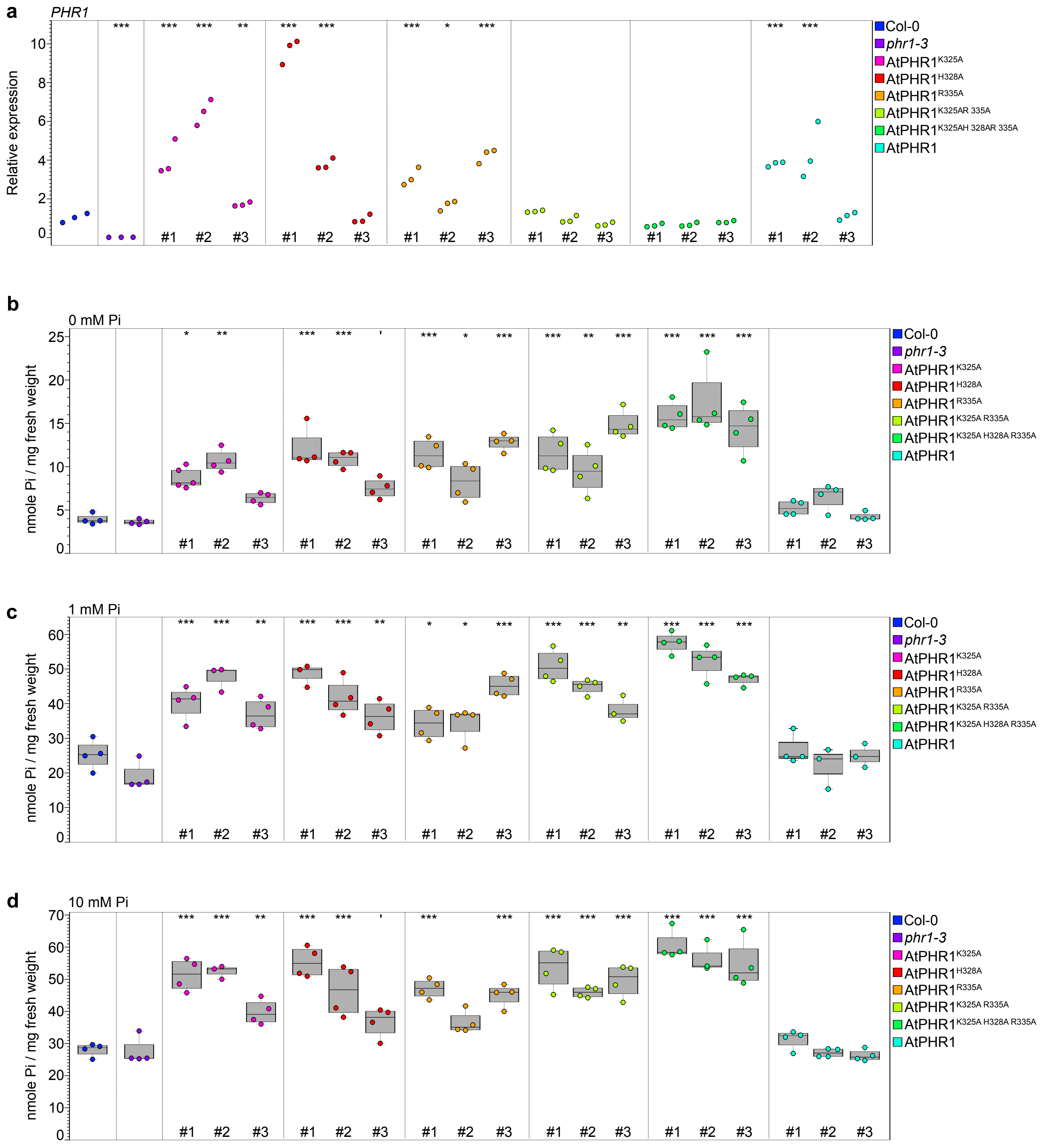

Supplementary Fig. 8 Mutations in the AtPHR1 KHR motif result in Pi hyper-accumulation.

a Expression of *PHR1* in Col-0, *phr1-3*, and seedlings of *phr1-3* complementation lines expressing FLAG-AtPHR1, FLAG-AtPHR1^{K325A}, FLAG-AtPHR1^{H328A}, FLAG-AtPHR1^{R335A}, FLAG-AtPHR1$^{K325A\ R335A}$, and FLAG-AtPHR1$^{K325A\ H328A\ R335A}$ under the control of the *AtPHR1* promoter relative to the housekeeping gene *Actin2* at 14 DAG. Seedlings were germinated and grown on vertical $^{1/2}$MS plates for 8 d, transferred to $^{1/2}$MS plates supplemented with 1 mM Pi and grown for additional 7 d. For each line, three biological replicates were analysed in technical triplicates by qRT-PCR. Stars indicate significant differences to Col-0 (*, $p < 0.05$; **, $p < 0.01$; ***, $p < 0.001$). **b-d** Plot represents Pi content of Col-0 wild type, *phr1-3* seedlings and seedlings of *phr1-3* complementation lines described in **a**. Bold black line, median; box, Interquartile range (IQR); whiskers, lowest/highest data point within 1.5 IQR of the lower/upper quartile. Seedlings were germinated and grown on vertical $^{1/2}$MS plates for 8 d, transferred to $^{1/2}$MS plates supplemented with either 0 mM (b), 1 mM (c) or 10 mM (d) Pi and grown for additional 7 d. For each line, 4 plants were measured in technical duplicates. (*, $p < 0.05$; **, $p < 0.01$; ***, $p < 0.001$).

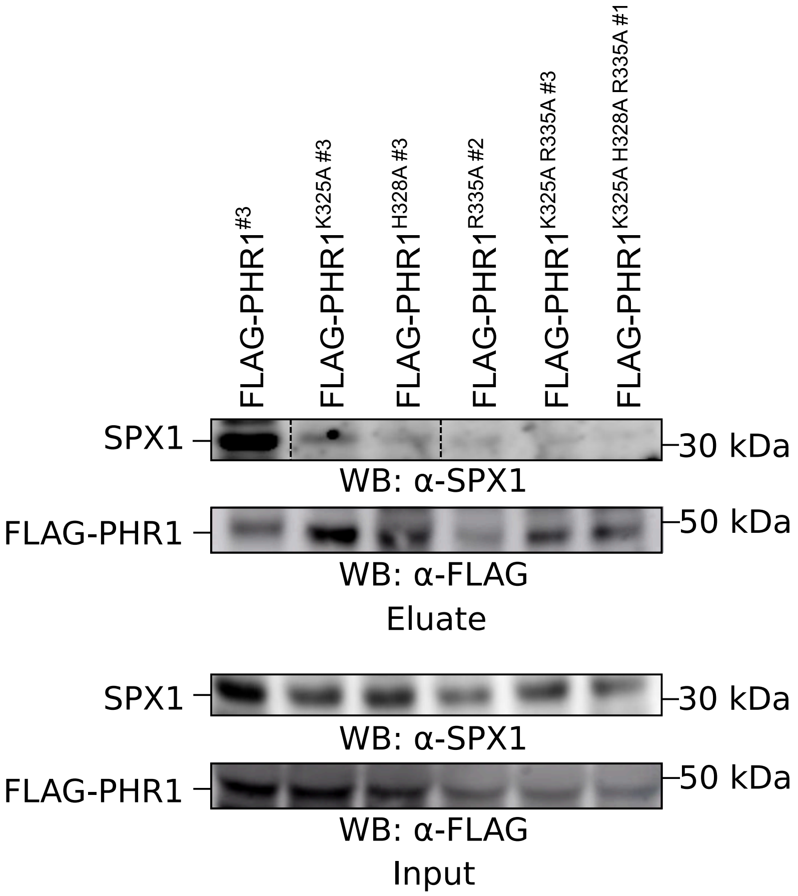

Supplementary Fig. 9 Mutation in the KHR motif reduces AtPHR1 binding to AtSPX1 in Arabidopsis.
Co-immunoprecipitation experiments using FLAG-tagged wild type and mutant AtPHR1 variants stably expressed in Arabidopsis under the control of the *AtPHR1* promoter. Total protein was extracted from *phr1-3* complementation lines at 10 DAG. Seedlings were germinated and grown on vertical $^{1/2}$MS plates supplemented with 1 mM Pi. FLAG-tag fusions were affinity bound with magnetic FLAG-tag trap, and immunoprecipitation of FLAG-AtPHR1 was monitored by immunoblot with an anti-FLAG antibody. Co-enrichment of endogenous AtSPX1 was monitored by immunoblot with an anti-SPX1 antibody.
