# Supplementary figures and images for: Inositol pyrophosphates promote the interaction of SPX domains with the coiled-coil motif of PHR transcription factors to regulate plant phosphate homeostasis
Source: Nat Commun. 2021 Jan 15;12:384. doi: 10.1038/s41467-020-20681-4 (PMC7810988; doi:10.1038/s41467-020-20681-4)

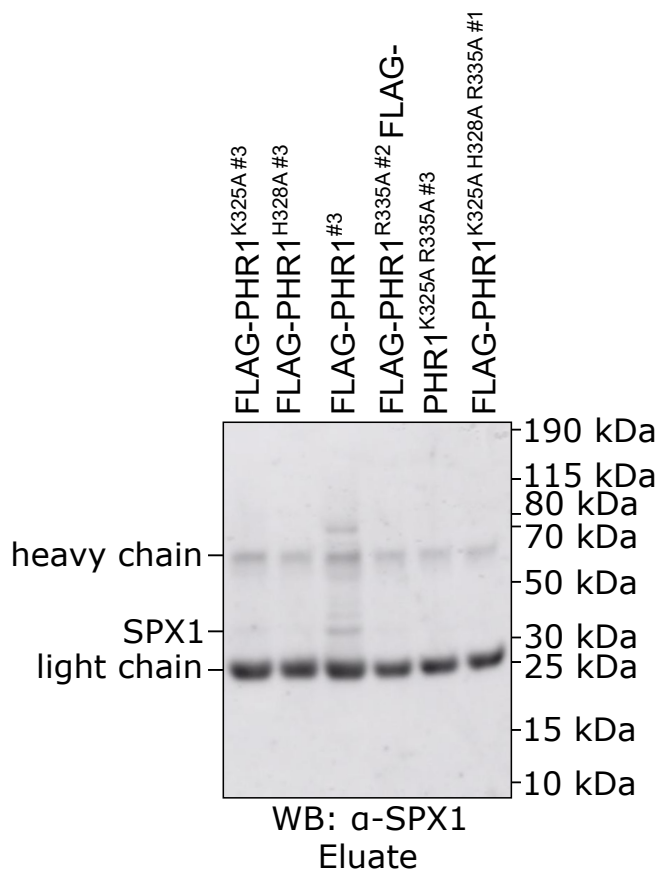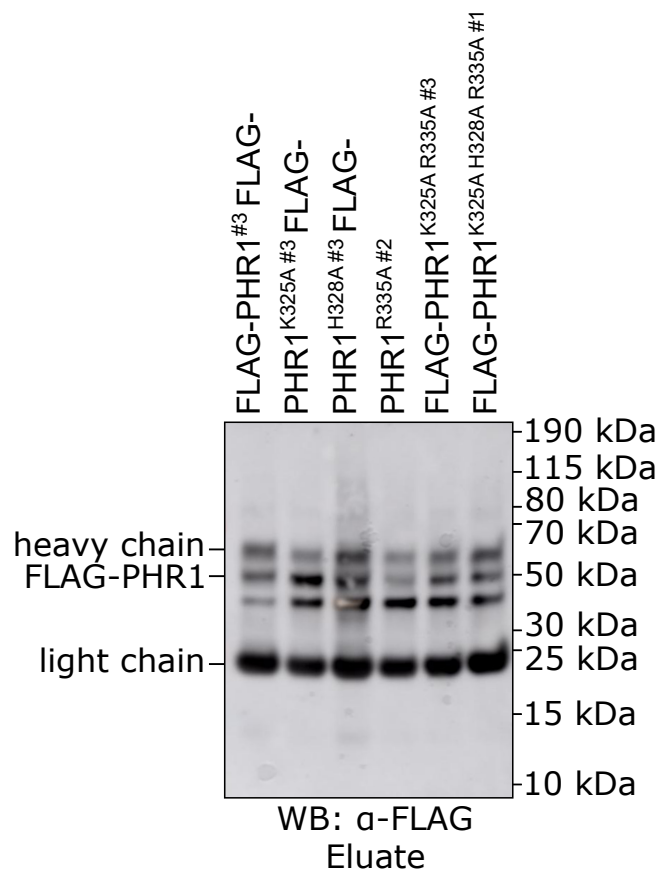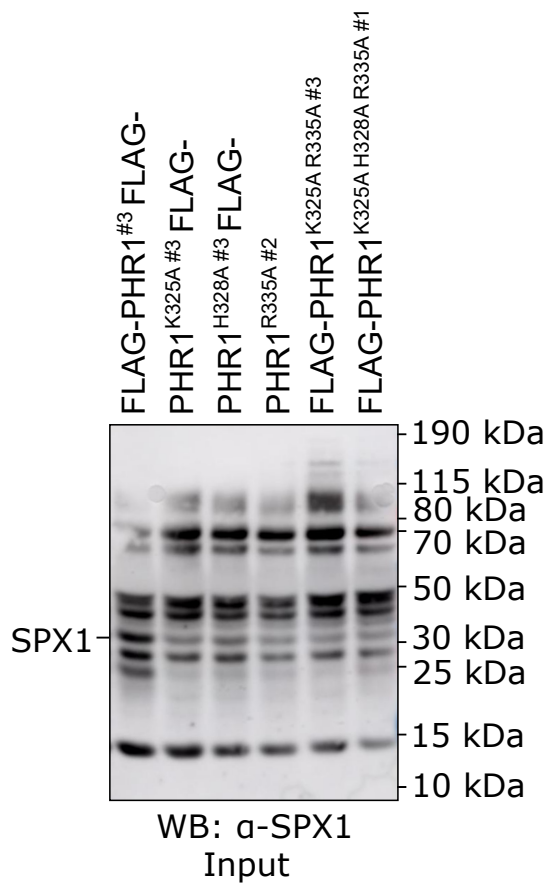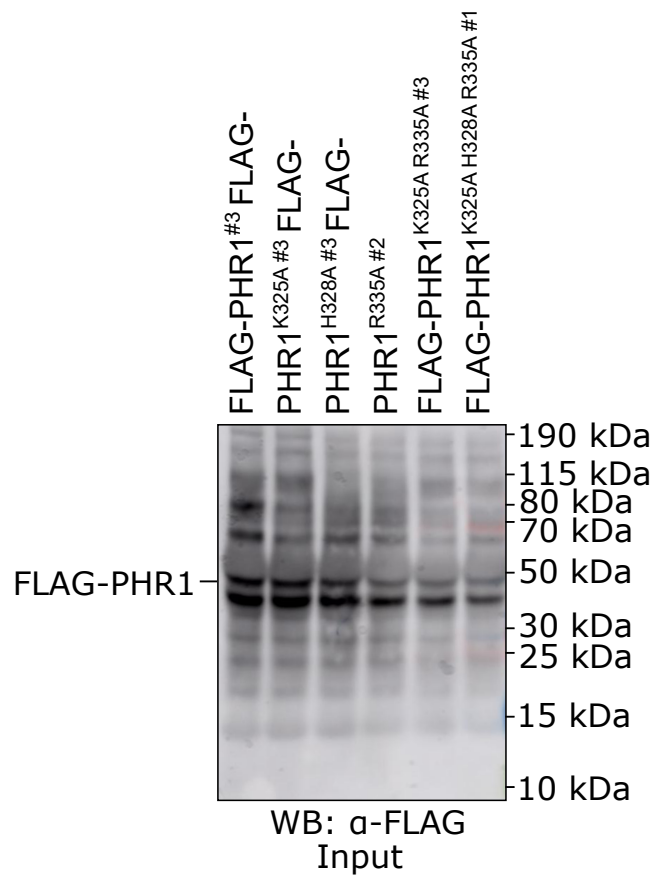

Supplement: Supplementary file 4 — Source Data [file 41467_2020_20681_MOESM4_ESM.zip › Ried_PHR_raw_data/fig_s9/uncropped_blots_fig_s9.pdf]

AtPHR1 222 - 358 Olig1

1.2  $\mu$ g

240 ng

48 ng

9.6 ng

1.92 ng

384 pg

76.8 pg

15.4 pg

complex →

free DNA →

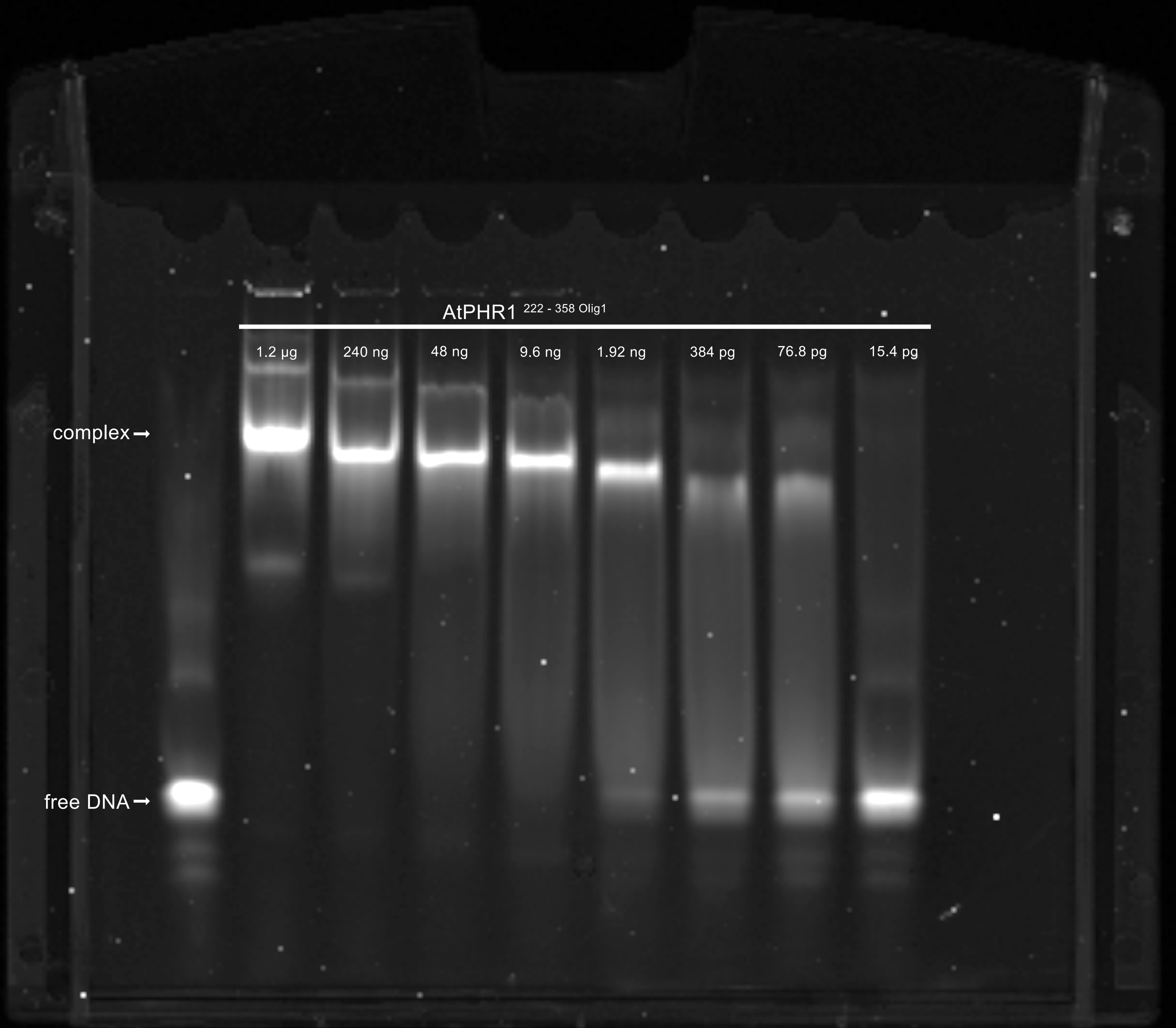

Supplement: Supplementary file 4 — Source Data [file 41467_2020_20681_MOESM4_ESM.zip › Ried_PHR_raw_data/fig_2c/AtPHR1_222_358_Olig1/AtPHR1_222_358_Olig1.pdf]

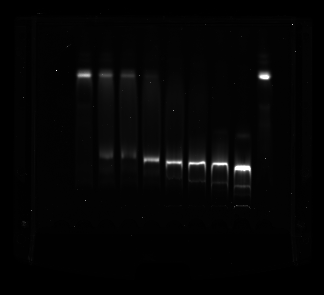

Supplement: Supplementary file 4 — Source Data [file 41467_2020_20681_MOESM4_ESM.zip › Ried_PHR_raw_data/fig_2c/AtPHR1_222_358_Olig1.tif]

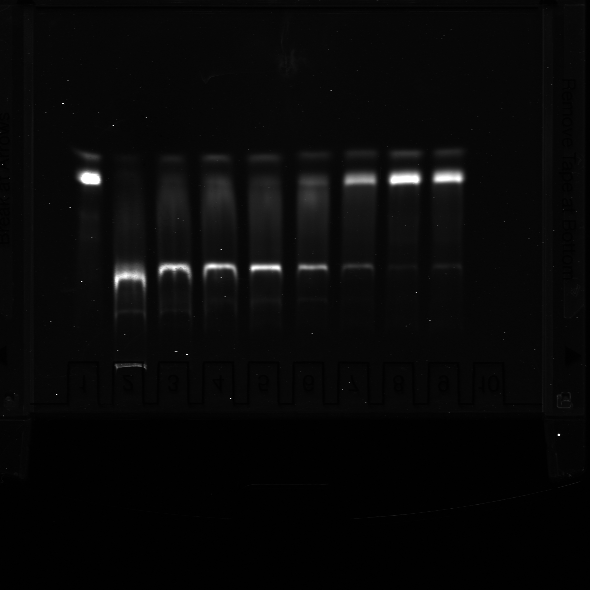

Supplement: Supplementary file 4 — Source Data [file 41467_2020_20681_MOESM4_ESM.zip › Ried_PHR_raw_data/fig_2c/AtPHR1_222_358_wt.tif]

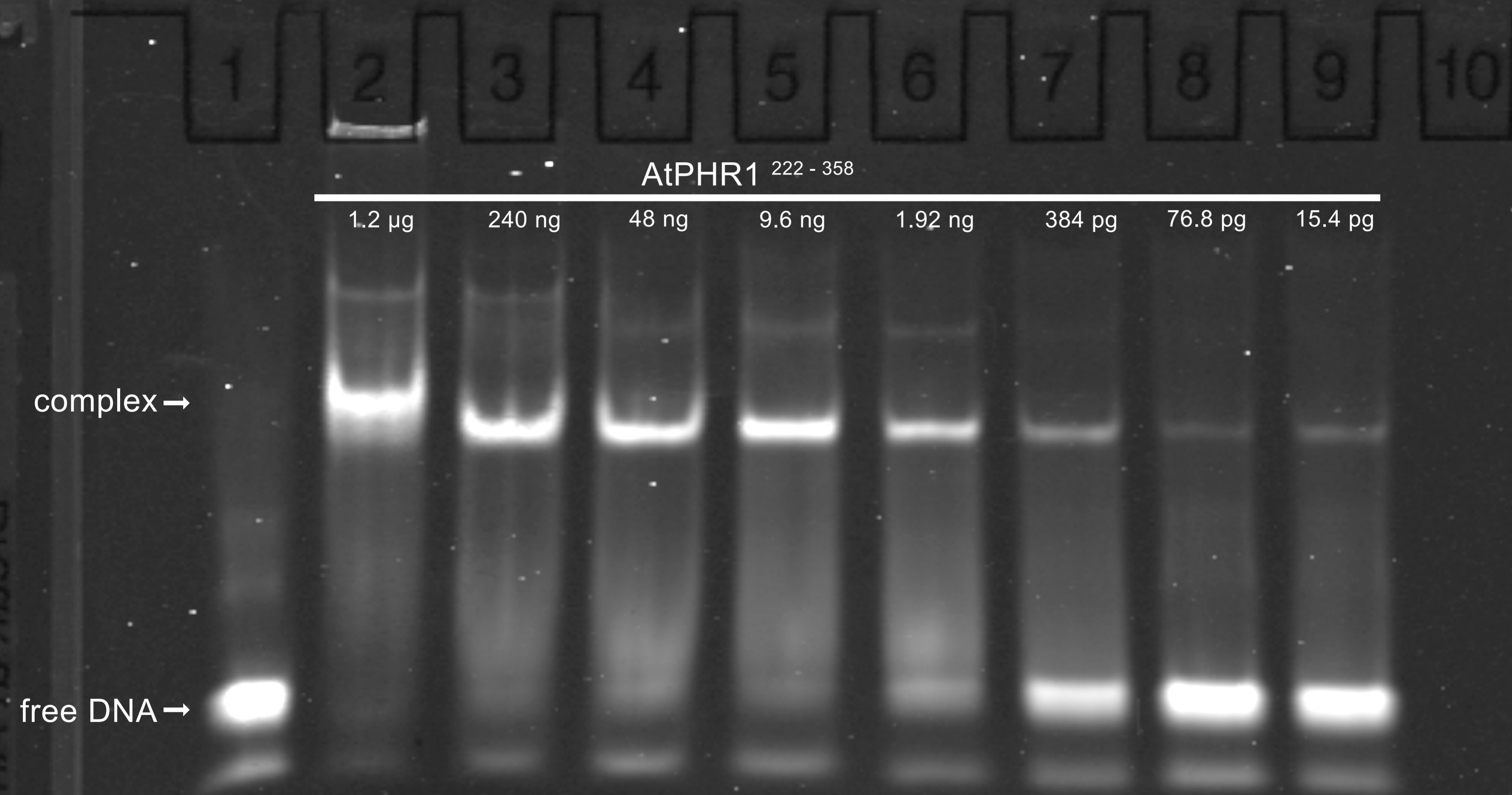

Supplement: Supplementary file 4 — Source Data [file 41467_2020_20681_MOESM4_ESM.zip › Ried_PHR_raw_data/fig_2c/AtPHR1_222_358_wt/AtPHR1_222_358_wt.pdf]

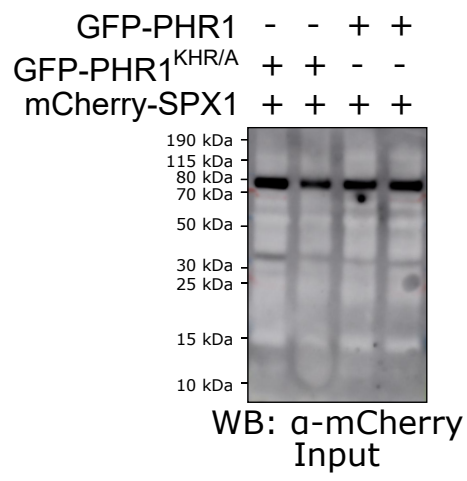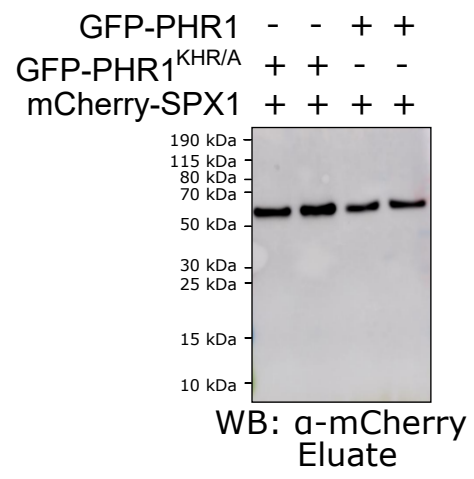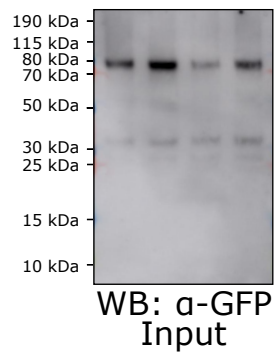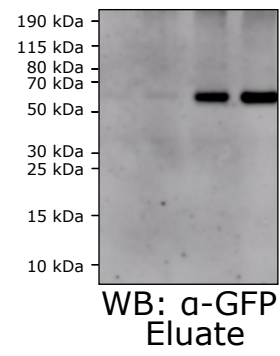

Supplement: Supplementary file 4 — Source Data [file 41467_2020_20681_MOESM4_ESM.zip › Ried_PHR_raw_data/fig_4d/uncropped_gels_fig_4d.pdf]
